# Does robotic assisted surgery mitigate obesity related systemic inflammatory response and clinical outcomes in left sided colorectal cancer resections?

**DOI:** 10.1007/s11701-025-02261-0

**Published:** 2025-03-05

**Authors:** Abigail R. Ingham, Stephen T. McSorley, Donald C. McMillan, David Mansouri, David Chong, Graham J. MacKay, Aleksandra Wrobel, Chia Yew Kong, Ahmed Alani, Gary Nicholson, Campbell S. D. Roxburgh

**Affiliations:** 1 Academic Unit of Surgery and School of Cancer Sciences, Glasgow, Scotland; 2 Queen Elizabeth University Hospital, NHS Greater Glasgow and Clyde, Glasgow, Scotland

**Keywords:** Robotic surgery, Obesity, Colorectal cancer, Rectal cancer, Inflammation, Complications, C-reactive protein, Systemic inflammatory response

## Abstract

Obesity (BMI > 30 kg/m^2^) is rapidly increasing worldwide with 26% of the UK population being obese and 38% being overweight. Obesity is intimately related to several life-limiting conditions including colorectal cancer (CRC). Obese patients have a higher degree of perioperative systemic inflammatory response (SIR) and an increased risk of perioperative complications. The aim of this current study was to investigate whether robotic-assisted surgery mitigates the effects of obesity in left sided CRC resections on the SIR and clinical outcomes. All patients undergoing left-sided colorectal cancer resections from May 2021 to May 2023 were, prospectively, entered into a database with patient characteristics and perioperative short-term outcomes recorded. CRP was considered a surrogate for SIR. The relationship between obesity and complications were examined using Chi Square for linear association, Kruskal–Wallis for continuous data and multivariate binary logistic regression model. 221 patients who underwent RAS for left-sided CRC were analysed. Obesity was associated with more comorbidity (ASA, *p* < 0.01) and SSI (*p* < 0.05) but not with age, sex, procedure or pathology. POD3 CRP < 150 mg/l was also associated with obesity (*p* < 0.01). In turn, greater comorbidity was associated with age (*p* < 0.001), site of resection (*p* < 0.05), SSI (*p* < 0.05), postoperative blood transfusion (*p* < 0.01) and LOS (*p* < 0.001). On multivariate analysis, only greater ASA (*p* < 0.05) and surgical procedure (*p* < 0.01) were associated with the development of an SSI independently. Greater comorbidity but not obesity was independently associated with postoperative SIR and clinical outcomes in patients undergoing RAS. These results support the use of RAS for left sided CRC resections, particularly in the obese.

## Introduction

Colorectal cancer is the 4th most common cancer in the UK [1] affecting an estimated 43,000 people per year, 54% of these new cases are thought to be preventable. The risk factors include poor dietary fibre intake, excessive red meat consumption, alcohol consumption, smoking, and obesity [2]. Obesity is classified as a body mass index (BMI) of 30 [3] or above and is becoming increasingly prevalent both in the UK and globally with 26% of the UK population currently obese and a further 38% overweight (BMI 25–30); in Scotland the problem is more marked with 30% in the obese category and an additional 37% in the overweight category [4]. At the core of treatment for most colorectal cancers is surgical resection which poses both an intraoperative technical challenge and is associated with a higher perioperative risk factor profile in the obese patient [5–9], demonstrated through longer operating time, greater frequency of surgical site infections (SSI), conversion to open surgery and anastomotic leak [10, 11].

Obesity is recognised to be associated with a number of metabolic and cardiovascular comorbidities as well as gastrointestinal cancers, partially due to its role in immune dysfunction [12]. In states of “overnutrition” or excessive adiposity, a phenotypic switch occurs in the adipocytes at the stromal level, releasing a cascade of circulating pro-inflammatory cytokines [13] which have a role in insulin dysregulation and glucose uptake, which in effect becomes a disease state of chronic inflammation. An increased degree of systemic inflammatory response has a multitude of implications for homeostatic, hepatic, neuroendocrine and metabolic functioning.

Although the degree of SIR generated is multifactorial and ultimately associated with the degree of trauma and intervention, prior studies demonstrate increased BMI and obesity are independent factors in the magnitude of this response [14]. Any form of surgical intervention or injury is associated with immediate acute local tissue effects brought about by pro-inflammatory mediators and innate immune activation. Local release of pro-inflammatory cytokines are responsible for hepatic production of C-reactive protein (CRP) leading the SIR in response to trauma/surgery [15–17]. CRP is widely considered a validated prototypical marker of systemic inflammation regardless of how it occurs. There is a proven relationship between CRP and postoperative outcomes and it used routinely in clinical practice, which lends itself to the observational and prospective nature of this study. Thus CRP can be considered a surrogate for the magnitude of the systemic inflammatory response in patients undergoing colorectal resection for left sided cancers, and prognostic factor in relation to oncological outcomes [18–22]. Although there are other validated markers of inflammation such as IL-6 etc., these are not clinically routinely measured [19, 20].

Following abdominal surgery, the magnitude of the SIR is usually predictable with peak levels of CRP seen on postoperative days 2 or 3. In colorectal surgery, previous research has identified thresholds for CRP on D2 or D3, with levels > 150 mg/l providing a risk assessment for the potential risk of developing infective complications [23, 24]. Due to the multi-system impact of the SIR, it has previously been hypothesised that high levels of perioperative inflammation contribute to the increased risk of developing complications and is a negative predictive factor in clinicopathological and oncological outcomes [25–27]. Therefore, considerable efforts are made to minimise the systemic inflammatory response after surgical resection. For example, the use of preoperative corticosteroids as part of the anaesthetic induction is recognised to reduce the perioperative SIR is [28, 29]. Minimally invasive surgery (MIS) [30] and in particular robotic assisted surgery (RAS) in left sided colorectal cancer resections [31] is thought to reduce the SIR. Previous studies have confirmed non-inferiority of RAS vs laparoscopic surgery (LS) with RAS having similar short-term outcomes, less systemic inflammation, less blood loss and shorter length of hospital stay [32, 33].

A previous study within this institute demonstrated RAS was an independent factor in the reduction of length of inpatient hospital stay, perioperative complication profile and postoperative CRP levels when compared to a matched cohort of laparoscopic and open left sided colorectal cancer resections [31]. Continuing on from this study and in a larger multi-site cohort, the impact of obesity as a factor in the development of perioperative complications and SIR is explored following robotic colorectal resection.

It is hypothesized that obesity would remain a significant factor in the development of perioperative complications and a higher magnitude systemic inflammatory response in patients having robotic resections. This is based on previous literature in open and laparoscopic surgery confirming higher rates of complications in those with BMI > 30kg/m^2^. In this present study, we investigate the role of obesity in the development of perioperative inflammatory responses and short-term outcomes in left sided colorectal robotic surgery.

### Methods and patients

Between May 2021 and May 2023 all patients at Glasgow Royal Infirmary (GRI) undergoing robotic surgical resection of colorectal cancer have been prospectively added into a departmental electronic database. Since the introduction of the robotic console (Da Vinci Xi model), all suitable patients with left sided colorectal cancer have been directed towards robotic surgery as the modality of choice. Demographic and clinical outcome data have been recorded for audit and research purposes including BMI, sex, 30-day postoperative complications and postoperative CRP results.

In the phase 1 roll out of robotics in Scotland, only left sided resections were performed robotically. Resections (referred to as Surgical Procedure in tables and text) include high anterior resection with anastomosis above peritoneal reflection, including upper rectal > 12cm from anorectal ring, sigmoid and distal colon cancers (HAR), low anterior resection (LAR) for rectal cancers, abdominoperineal resections (APR) and “other” procedures which include subtotal and panproctocolectomy.

Tumour pathology was graded using the TNM classification as described by the Royal College of Pathologists [34].

The usage of intraoperative dexamethasone and the dosage was at the discretion of the anaesthetist, doses varying 3.3 mg to 8 mg.

Body mass index (BMI) was categorised per the WHO BMI classes: < 18.5 kg/m^2^ (underweight), 18.5–24.9 kg/m^2^ (normal weight), 25–29.9 kg/m^2^ (overweight) and ≥ 30 kg/m^2^ (obese). Within the obese category this was further divided into obesity class 1 (30–34.9 kg/m^2^) and obesity class 2/3 (≥ 35 kg/m^2^) per BMI obesity classes. Underweight patients were excluded from analysis due to the different inflammatory profiles associated with cancer cachexia [35].

The perioperative SIR was measured using serum CRP (mg/l) locally and analysed using an auto-analyser (Architect; Abbot Diagnostics) with the same lower detectable limit of 0.2 mg/l throughout the study. A result above 9mg/l is considered raised. It is routine practice for patients to attend a preoperative assessment where CRP level is checked, and again postoperatively on days 1–4 or until deemed clinically appropriate/ discharge from hospital. The threshold of < 150 mg/l was used as a cohort variable based on previous studies as a safe value for discharge with decreased likelihood of a complication occurring. All patients were enrolled in an established ERAS programme and had mechanical and oral antibiotic bowel preparation.

Complications were graded using the Clavien–Dindo (CD) classification where complications range from CD0 (no complication) to CD5 (mortality within 30 days). Major complications were defined as CD3-5 which included reoperation within 30 days and unplanned admission to level 2/3 care for single or multiple organ system dysfunction [36].

### Statistical analysis

Clinical and pathological data were categorised per standard groupings/thresholds. Categorical data were reported as patient numbers and proportions within each of the BMI cohorts accordingly. The χ2 test or Fisher’s exact test was used to analyse categorical variables.

Median values were used to describe continuous variables and differences between surgical approach and BMI categories were compared using Kruskal–Wallis test.

A univariate model and multivariate binary logistic regression model were applied to examine the relationship between the development of postoperative SSI and key clinico-pathological variables. Odds ratios were presented with 95% confidence intervals. Variables with significant *P* Values on univariate analysis were included into the multivariate binary logistic regression model. A backwards conditional model was used.

A statistically significant P value was defined as *P* < 0.05.

All statistical analyses were executed using SPSS Version 29.

## Results

221 patients who underwent RAS for a left sided CRC were analysed. The majority of these patients were overweight (*n* = 176, 79%) and of these 83 (37%) were obese. The most commonly performed surgical procedure was HAR, followed by LAR and APR.

 The patient characteristics and perioperative outcomes per BMI category are shown in Table 1. Obesity was associated with more comorbidity (ASA, *p* < 0.01) and SSI (*p* < 0.05) but not with age, sex, procedure type or pathology. Obesity was conversely associated with POD 3 < 150 mg/l (*p* ≤ 0.01). There was no significant association between greater obesity and the rate of minor or major/CD3 + complications, length of hospital stay or 30 day readmission to hospital rate. No patients undergoing colorectal RAS died within 30 days of operation or index admission.

**Table 1:** Perioperative outcomes per BMI category in left sided RAS colorectal cancer resections

Outcomes	BMI 18.5–24.9 kg/m2 (N = 45)	BMI 25–29.9 kg/m2 (N = 93)	BMI 30- 34.9 kg/m2 (N = 50)	BMI ≥ 35 kg/m2 (N = 33)	P value
Age ≤ 54 Age 55–74 Age 75	8 (17.8%)	16 (17.2%)	11 (22%)	9 (27.3%)	0.865
	27 (60%)	57 (61.3%)	31 (62%)	19 (57.6%)	
	10 (22.2%)	20 (21.5%)	8 (16%)	5 (15.2%)	
Sex Male	24 (53.3%)	56 (60.2%)	30 (60%)	18 (54.5%)	0.842
Surgical Procedure: High Anterior Low Anterior APR Other	22 (48.9%)	42 (45.2%)	23 (46%)	14 (42.4%)	0.501
	15 (33.3%)	34 (36.6%)	21 (42%)	10 (30.3%)	
	6 (13.3%)	13 (14%)	6 (12%)	9 (27.3%)	
	2 (4.4%)	4 (4.3%)	0	0	
ASA ≥ 3	13 (28.9%)	25 (27.2%)	21 (42%)	19 (59.4%)	**0.006**
≥ T3 disease	28 (65.1%)	50 (54.3%)	27 (54%)	19 (61.3%)	0.609
Node positive	8 (20%)	25 (27.5%)	17 (36.9%)	6 (20%)	0.495
Metastatic disease	1 (2.3%)	5 (5.4%)	0	2 (6.3%)	0.317
Intraoperative Dexamethasone	37 (86%)	75 (88.2%)	40 (87%)	26 (83.9%)	0.939
Conversion	2 (4.4%)	3 (3.2%)	1 (2%)	0	0.655
Preop CRP ≥ 10 mg/l	7 (19.4%)	16 (18.6%)	6 (13.3%)	4 (17.4%)	0.871
Median POD 1 CRP mg/l	48	46	53	51	0.561
Median POD 2 CRP mg/l	102	81	78	96	0.255
Median POD 3 CRP mg/l	110	87	83	87	0.083
Median POD 4 CRP mg/l	86	69	65	106	**0.035**
POD 3 CRP < 150 mg/l	21 (60%)	62 (75.6%)	42 (91.3%)	22 (71%)	**0.011**
Median LOS (days)	7	6	6	7	0.665
AL	2 (4.4%)	3 (3.2%)	0	0	0.350
SSI	9 (20.5%)	5 (5.4%)	4 (8%)	5 (15.2%)	**0.047**
Infective complication	9 (20.5%)	13 (14.9%)	8 (17.8%)	6 (20%)	0.726
HAP	1 (2.2%)	5 (5.4%)	1 (2%)	1 (3%)	0.868
PO Blood transfusion	4 (9.1%)	4 (4.3%)	1 (2%)	0	0.189
CD ≥ 3 complications	3 (6.7%)	6 (6.5%)	5 (10%)	2 (6.1%)	0.863
Readmission to hospital within 30 days	1 (2.2%)	9 (9.8%)	5 (10%)	4 (12.1%)	0.373

Bold values indicates *p* < 0.005

The patient characteristics and perioperative outcomes per ASA category are shown in Table 2. Greater comorbidity was significantly associated with age (*p* < 0.001), site of resection (*p* < 0.05), SSI (*p* < 0.05), postoperative blood transfusion (*p* < 0.01) and LOS (*p* < 0.001).

**Table 2:** Characteristics and outcomes for patients undergoing left sided colorectal cancer resection per ASA cohort

Outcomes	ASA 1–2 (n = 141)	ASA 3–4 (n = 78)	P value
Age ≤ 54 Age 55–74 Age 75	38 (27%)	6 (7.7%)	** < 0.001**
	83 (58.9%)	50 (64.1%)	
	20 (14.2%)	22 (28.2%)	
Sex: male	80 (56.7%)	47 (60.3%)	0.613
Surgical procedure: high Anterior Low anterior APR Other	68 (48.2%)	33 (42.3%)	**0.013**
	56 (39.7%)	23 (29.5%)	
	13 (9.2%)	20 (25.6%)	
	4 (2.8%)	2 (2.6%)	
Obesity (BMI ≥ 30 kg/m^2^)	42 (29.8%)	40 (51.3%)	**0.002**
≥ T3 disease (0/1)	77 (56.6%)	47 (60.3%)	0.604
Node positive (0/1)	33 (26%)	21 (28.4%)	0.708
Metastatic disease (0/1)	6 (4.3%)	2 (2.6%)	0.511
Intraoperative Dexamethasone (0/1)	120 (90.2%)	58 (80.6%)	0.051
Conversion (0/1)	3 (2.1%)	3 (3.8%)	0.456
Preop CRP ≥ 10 mg/l	23 (18.1%)	10 (16.4%)	0.772
Median POD 1 CRP mg/l	50 (3–210)	43 (10–173)	0.249
Median POD 2 CRP mg/l	82 (16–477)	92 (15–415)	0.824
Median POD 3 CRP mg/l	87 (15–472)	96 (15–452)	0.562
Median POD 4 CRP mg/l	70 (12–412)	96 (13–519)	0.113
POD 3 CRP < 150 mg/l	94 (76.4%)	52 (75.4%)	0.869
Median LOS (days)	5	8	** < 0.001**
Anastomotic Leak	3 (2.1%)	2 (2.6%)	0.836
SSI	8 (5.7%)	15 (19.2%)	**0.002**
Infective complication	14 (11%)	14 (21.5%)	0.051
Hospital Acquired Pneumonia	3 (2.1%)	5 (6.4%)	0.106
PO Blood transfusion	2 (1.4%)	7 (9%)	**0.007**
CD ≥ 3 complications	8 (5.7%)	8 (10.3%)	0.212
Readmission to hospital within 30 days	10 (7.1%)	9 (11.7%)	0.250

Bold values indicates *p* < 0.005

The relationship between perioperative factors and the development of SSI are shown in Table 3. When using univariate analysis, ASA (*p* < 0.01), surgical procedure (*p* < 0.001),  ≥ T3 disease, node positive disease (*p* < 0.05) and intra-operative dexamethasone (*p* < 0.05) were significant associated with SSI. On multivariate analysis, only greater ASA (*p* < 0.05) and surgical procedure (*p* < 0.01) were independently associated with the development of an SSI.

**Table 3:** The relationship between surgical site infection rate and preoperative patient characteristics in those undergoing left sided colorectal cancer resections

SSI	Univariate	Multivariate
Age	0.99 (0.96–1.003) p = 0.618	
Sex	0.76 (0.32–1.81) p = 0.538	
ASA grade	**3.93 (1.58–9.75) p = 0.003**	**3.52 (1.23–10.09) p = 0.019**
Surgical procedure	**2.49 (1.50–4.14) p < 0.001**	**2.27 (1.26–4.09) p = 0.007**
Obesity (BMI ≥ 30 kg/m^2^)	1.07 (0.44–2.59) p = 0.883	
≥ T3 disease (0/1)	**0.38 (0.15–0.95) p = 0.038**	0.406 (0.14–1.17) p = 0.096
Node positive (0/1)	**0.15 (0.02–0.98) p = 0.048**	0.16 (0.02–1.25) p = 0.081
Metastatic disease (0/1)	3.00 (0.57–15.81) p = 0.195	
Intraoperative Dexamethasone (0/1)	**0.35 (0.12–0.99) p = 0.047**	0.31 (0.09–1.11) p = 0.071
Conversion (0/1)	1.74 (0.20–15.62) p = 0.618	
Preop CRP ≥ 10 mg/l	1.53 (0.47–5.02) p = 0.485	

Bold values indicates *p* < 0.005

## Discussion

It was anticipated that obese patients undergoing RAS for left sided colorectal cancer would have poorer clinical outcomes and demonstrate a significantly higher systemic inflammatory response. Contrary to the above hypothesis, this present study demonstrated obesity was not a significant factor in the development of infective complications following RAS. Although obesity was associated with greater preoperative comorbidity and post-operative systemic inflammation this did not translate to less favourable outcomes; only greater comorbidity (ASA3 +) was independently associated with poorer clinical outcomes, in particular SSI. It is therefore considered that RAS could be a factor in dampening the recognised inflammatory effects of obesity.

Obesity is intimately related to multiple comorbid conditions that can affect the perioperative systemic inflammatory response and clinical outcomes. Associated conditions include type 2 diabetes mellitus, coronary heart disease, atherosclerotic disease as well as respiratory compromise through obstructive sleep apnoea and excessive weight on the chest wall. It is therefore perhaps unsurprising that the obese cohort in the present study had higher proportions of ASA 3 and 4 scores. Indeed, it has been reported that patients with a BMI ≥ 30 may be considered to be a minimum of ASA grade 2 [37] and ASA 3 and above are recognised to be independently associated with increased likelihood of major (CD3 +) complications [38–40]. In this current study, greater ASA and type of surgical procedure, but not obesity, was significantly associated with the development of SSI. Given that all patients were subject to the same entry criteria and perioperative care it may that RAS uncouples the relationship between obesity and SSI. Indeed, there is good evidence that SSI rates are lower in minimally invasive surgery compared with open surgery. It may be that RAS lowers this further in the obese patient. Irrespective, given the increasing proportion of obese patients with presenting with colorectal cancer the relationship with SSI and mitigating its effect is of considerable interest.

Several biochemical mechanisms have been hypothesized as to how obesity is linked to increased risk of SSI such as decreased oxygen circulation within the tissues, collagen synthesis deficiencies, immune dysfunction and inadequate infiltration of intravenous antibiotics to the wound/surgical site [41]. In addition, there are technical aspects of operating on the obese such as increased depth and length of wound, increased wound dead space between skin and sheath with often long operating times due to difficult access and visibility secondary to increased visceral fat. While some studies have shown robotic surgery to be beneficial in terms of mortality [42], reduced LOS [43] and reduced conversion rate [44, 45] for the obese in left sided colorectal cancer resections, to our knowledge this is the first study to examine the relationship between obesity and SIR in the robotic platform.

RAS is thought to reduce the surgical trauma through low pressure pneumoperitoneum (7mmHg vs 12-15mmHg traditionally used in MIS), wristed instruments allowing better manoeuvrability, operator controlled 3d binocular camera allowing better visualisation with smaller incisions [46]. In APRs, a substantial perineal wound is often required to achieve oncological safety and to allow appropriate visualisation, especially in the narrow obese male pelvis. The RAS console allows for angulated views with fixed retraction of tissues down in the pelvis which means a higher proportion of the rectal dissection can be performed intracorporeally, warranting a smaller perineal wound and possibly avoiding the requirement of plastic surgery reconstruction [47]. This improved visualisation is evident in all left sided resections, which may explain the significantly reduced conversion rate across the board when compared with other MIS techniques [48, 49]. The smaller incisions and less frequent regrasping and crushing of tissues may contribute to the perceived reduced surgical stress response with less stretch on the peritoneum from the lower pressure environment and less counterpressure to the diaphragm. In the present study, consistent with the above, it was of interest that the surgical procedure was independently associated with SSI.

To compare the effects of obesity and ASA groupings on SSI rate, Table 4 was formulated. In the whole study population, there was an approximately three-fold greater rate of SSI in the comorbid patients (19.2% vs 5.7%, *p* = 0.02) but when analysis was stratified by BMI this was largely confined to the normal BMI population (23.7% vs 5.1%, *p* = 0.001). The SSI rate was approximately 10% whereas pre-RAS studies from our institution reported SSI rates of approximately 20% in obese patients (approximately 29% of historic cohort were obese compared with 37% in the present cohort) using open and laparoscopic surgery [5]. Therefore, it is clear that against a background of increasing obesity, SSI rates are lower in patients undergoing RAS for colorectal cancer. It is therefore proposed that all obese patients should be directed towards robotic assisted colorectal surgery for resection of left sided cancers to minimise the SIR and lower rates of SSI.

**Table 4:** The relationship between obesity and ASA category versus the rate of 30 day surgical site infection

SSI Rate	ASA 1–4 (N = 221)	ASA 1&2 (N = 141)	ASA 3&4 (N = 78)	P value (ASA 3&4 vs SSI)
BMI < 30 kg/m^2^ (N = 138)	14 (10.2%)	5 (5.1%)	9 (23.7%)	**0.001**
BMI ≥ 30 kg/m^2^ (N = 83)	9 (10.8%)	3 (7.1%)	6 (15%)	0.255
ALL BMI (N = 221)	23 (10.5%)	8 (5.7%)	15 (19.2%)	**0.002**
P Value (BMI > 30 vs SSI)	0.883	0.634	0.331	

Bold values indicates *p* < 0.005

A limitation to this study could be considered the use of only robotic cases. Although it would be possible to compare with a historic cohort of open or laparoscopic cases, it was felt this would not reflect current operative practice (including anaesthesia and post-operative practice, national move towards RAS for left sided colorectal cancer resection in 2021) and therefore introduce other potential confounding factors.

In summary, greater comorbidity but not obesity per se was independently associated with post-operative SIR and clinical outcomes in patients undergoing RAS. These results would support the use of RAS for left sided CRC resections, in particular in the obese.

## Data Availability

No datasets were generated or analysed during the current study.
